# Supplementary material for: Age and Comorbidities as Risk Factors for Severe COVID-19 in Mexico, before, during and after Massive Vaccination
Source: Vaccines (Basel). 2023 Nov 2;11(11):1676. doi: 10.3390/vaccines11111676 (PMC10674414; doi:10.3390/vaccines11111676)

Table S1. Published studies included in the review section. All contain data from before anti-SARS-CoV-2 vaccination in Mexico.

First Author (reference number)	Data from	Start and end of study period dd-mm-yy	N Total patients	N of SARS-CoV-2 Negative	N of confirmed as COVID-19 by RT-PCR	COVID-19 Cases					
						% male<br>% female	Ages included (average or median)	% Hospi-talized	%ICU	%Died	%with comorbidity
[8]	*475 USMER*	*27-02-20*<br>*30-04-20*	*23,593*	*18,443*	*3,844*	*58.0*<br>*42.0*	*All (45.4±15.8)*	19.0	12.7	8.8	56.0
[26]	*Open data-DGE SS*	*27-02-20*<br>*30-04-20*	*87,372*	*52,628*	*19,224*	*58.2*<br>*41.8*	*0-104 (46)*	39.4	NA	9.7	ND
[13]	*Open data-DGE SS*	*27-02-20*<br>*23 04 20*	*10,544*	*NA*	*10,544*	*57.8*<br>*42.2*	*All (46.5±15.2)*	37.9	4.7	9.2	44.3
[27]	*Open data-DGE SS*	*28-02-20*<br>*27-04-20*	*71,103*	*55,604*	*15,529*	*57.0*<br>*43.0*	*All (S 40, NS 60)*	NA	NA	16.1	17.6
[30]	*IMSS*	*28-02-20*<br>*28-03-20*	*740*	*NA*	*740*	*57.3*<br>*42.7*	*>15 (43.7±14.9)*	27.9	NA	NA	ND
[14]	*Open data-DGE SS*	*01-01-20*<br>*25-04-20*	*13,842*	*NA*	*13,842*	*57.7*<br>*42.3*	*All (46.6±15)*	38.8	2.4	9.4	45.3
[15]	*Open data-DGE SS*	*24-02-20*<br>*26-04-20*	*71,103*	*46,960*	*15,529*	*57.8*<br>*42.2*	*All (46.6)*	38.9	1.6-4.4	9.2	45.8
[25]	*Open data-DGE SS*	*21-02-20*<br>*28-04-20*	*16,752*	*NA*	*16,752*	*58.0*<br>*42.0*	*All (46.5±15.5)*	39.2	4.3	10.3	45.3
[28]	*Open data-DGE SS*	*28 02 20*<br>*15-05-20*	*32,583*	*20,279*	*12,304*	*58.7*<br>*41.3*	*All (45±11)*	45.0	4.2	12.2	ND
[20]	*Open data-DGE SS*	*27-02-20*<br>*18-05-20*	*51,053*	*NA*	*51,053*	*57.6*<br>*42.4*	*All (46.6±15.8)*	NA	NA	10.3	45.8
[17]	*Open data-DGE SS*	*27-02-20*<br>*18-05-20*	*177,133*	*98,567*	*51,633*	*57.7*<br>*42.3*	*All (46.6)*	38.4	3.7	10.3	ND
[10]	*12 third level hospitals*	*30-04-20*<br>*20-05-20*	*400*	*NA*	*400*	*70.0*<br>*30.0*	*Adults 20+ (NR)*	100	NA	50.0	ND
[11]	*INCMNSZ*	*16-03-20*<br>*21-05-20*	*329*	*NA*	*329*	*64.0*<br>*36.0*	*Adults 18+ (49±11)*	100	48.3	NA	70.0
[22]	*Open data-DGE SS*	*27-02-20*<br>*23-05-20*	*11,405*	*9,962*	*1,443*	*48.0*<br>*52.0*	*Children <18 (12)*	ND	3.3	1.9	44.4
[16]	*Open data-DGE SS*	*27-02-20*<br>*27-06-20*	*211,003*	*NA*	*211,003*	*54.7*<br>*45.3*	*All (45.7±16.3)*	31.0	2.6	12.3	47.4

[18]	*Open data-<br>DGE SS*	*13-01-20<br>17-07-20*	*331,298*	*NA*	*331,298*	*54.0<br>46.0*	*All (44±11)*	28.8	8.2	11.5	ND
[12]	*Open data-<br>DGE SS*	*01-06-20<br>23-07-20*	*492,160*	*308,381*	*183,779*	*54.0<br>46.0*	*All (46.3±15.9)*	26.5	2.4-3.3	7.9	ND
[19]	*IMSS*	*04-03-20<br>15-08 20*	*66,123*	*NA*	*66,123*	*60.6<br>39.4*	*Adults 20+ (53±14.5)*	100	NA	48.3	DM 33<br>HT 40.4
[21]	*Open data-<br>DGE SS*	*13-01-20<br>02-11-20*	*638,782*	*NA*	*638,782*	*51.4<br>48.6*	*All (44.4±16.7)*	25.6	5.6	11.0	43.0
[23]	*3 hospitals in<br>Mexico City:<br>HFMS, HGM,<br>HCMM*	*01-04-20<br>30-05-20*	*377*	*NA*	*377*	*73.1<br>26.9*	*Adults_18+<br>(S 41,5±22.7<br>NS 46,5±25,7)*	100	NA	20.9	56.0
[29]	*Open data-<br>DGE SS*	*01-03-20<br>01 02 21*	*608*	*NA*	*608*	*65.5<br>34.5*	*Adults 18+<br>(S 51± 10<br>NS 62± 10,2)*	100	23.0	43.4	100
[31]	*IMSS,*	*25-03-20<br>07-09-20*	*773*	*NA*	*773*	*62.7<br>37.3*	*Adults 18+<br>(OB 50±12)*	100	20-26	35.8	82.0
[24]	*HGM*	*30-11-20<br>09-07-21*	*378*	*NA*	*378*	*36.4<br>63.6*	*Adults 18+<br>(S 51,4±13,2<br>NS 58,8±13,7)*	100	37.8	32.5	ND

USMER=Unidades monitoras de enfermedad respiratoria (units that monitor repiratory disease); DGE SS= Dirección general de epidemiología (General Directory of Epidemiology) Secretaría de Salud (Health secretariat); IMSS= Instituto Mexicano del Seguro Social; INCMNSZ= Instituto Nacional de Ciencias Médicas y Nutrición Salvador Zubirán, HFMS= Hospital Fundación Médica Sury; HGM=Hospital General de México; HCMM=Hospital Central Militar; S= survivors; NS= non survivors; OB= mean age in obese individuals; NA= not analyzed; ND= no data.

Table S2. Symptomatic adult COVID-19 cases and deaths analyzed per wave, age and comorbidity in Mexico.

Age (years)	Comor-bidity	Wave 1 (Feb 16 2020-Sept 19 2020)		Wave 2 (Sept 20 2020-May 15 2021)		Wave 3 (May 16 2021-Nov 20 2021)		Wave 4 (Nov 21 2021-Abr 30 2022)		Wave 5 (May 1 2022-Oct 15 2022)		Wave 6 (Oct 16 2022-May 9 2023)		Total All waves (feb 2020-may 2023)	
		cases	deaths	cases	deaths	cases	deaths	cases	deaths	cases	deaths	cases	deaths	cases	deaths
**20-39**	no	202,831	1,992	492,791	2,575	596,909	3,272	750,143	445	507,199	134	160,309	68	**2,710,180**	**8,486**
	yes	85,609	3,339	155,430	3,759	131,459	3,707	133,034	660	84,038	126	35,380	78	**624,950**	**11,669**
	no+yes	288,440	5,331	648,221	6,334	728,368	6,979	883,177	1,105	591,237	260	195,689	146	**3,335,132**	**20,155**
	total	289,645	5,385	650,068	6,369	735,028	7,026	889,632	1,112	596,758	261	196,573	147	**3,357,704**	**20,300**
**40-59**	no	153,393	10,055	346,855	14,328	272,568	7,291	447,486	1,505	321,981	229	109,320	129	**1,651,603**	**33,537**
	yes	149,696	22,945	248,235	26,807	145,528	13,379	179,136	3,637	120,937	455	55,722	287	**899,254**	**67,510**
	no+yes	303,089	33,000	595,090	41,135	418,096	20,670	626,622	5,142	442,918	684	165,042	416	**2,550,857**	**101,047**
	total	304,589	33,278	597,004	41,360	422,116	20,782	631,490	5,169	447,343	686	165,807	416	**2,568,349**	**101,691**
**60-79**	no	41,214	11,802	92,241	19,583	59,170	7,449	73,854	3,052	74,519	491	30,486	337	**371,484**	**42,714**
	yes	92,990	36,069	160,096	51,869	79,975	19,792	70,952	8,591	58,211	1,413	31,965	1,042	**494,189**	**118,776**
	no+yes	134,204	47,871	252,337	71,452	139,145	27,241	144,806	11,643	132,730	1,904	62,451	1,379	**865,673**	**161,490**
	total	134,956	48,190	253,336	71,795	140,450	27,371	146,037	11,698	134,022	1,912	62,723	1,385	**871,524**	**162,351**
**80+**	no	5,732	2,694	12,397	5,113	7,927	2,345	9,247	1,949	7,927	468	4,218	322	**47,448**	**12,891**
	yes	15,148	8,273	27,432	13,500	14,136	5,722	14,534	4,620	9,453	1,039	6,649	783	**87,352**	**33,937**
	no+yes	20,880	10,967	39,829	18,613	22,063	8,067	23,781	6,569	17,380	1,507	10,867	1,105	**134,800**	**46,828**
	total	21,040	11,048	40,216	18,724	22,282	8,100	24,009	6,601	17,563	1,513	10,931	1,108	**136,041**	**47,094**
**All adults 20+**	no	403,170	26,543	944,284	41,599	936,574	20,357	1,280,730	6,951	911,626	1,322	304,333	856	**4,780,717**	**97,628**
	yes	343,443	70,626	591,193	95,935	371,098	42,600	397,656	17,508	272,639	3,033	129,716	2,190	**2,105,745**	**231,892**
	no+yes	**746,613**	**97,169**	**1,535,477**	**137,534**	**1,307,672**	**62,957**	**1,678,386**	**24,459**	**1,184,265**	**4,355**	**434,049**	**3,046**	**6,886,462**	**329,520**
	total	**750,230**	**97,901**	**1,540,624**	**138,248**	**1,319,876**	**63,279**	**1,691,168**	**24,580**	**1,195,686**	**4,372**	**436,034**	**3,056**	**6,933,618**	**331,436**

Mortality was significantly higher in older age groups, with p<0.0001 for all CFR comparisons of 40-59, 60-79, 80+ vs 20-39, per wave and in total. Mortality was significantly higher in wave 1-2 (before vaccines), *vs* wave 5 and/or 6 in every age adult group and in total adults, with p<0.0001.

Table S3. Risk factors associated with the development of pneumonia, the need for assisted mechanical ventilation or intensive care unit in COVID-19 from studies published before anti-SARS-CoV-2 vaccination (with data up to January 2021)

**Pneumonia**			**Assisted mechanical ventilation**			**Intensive Care Unit**		
**Risk factor**	**OR (95% CI)**	**Reference**	**Risk Factor**	**OR (95% CI)**	**Reference**	**Risk Factor**	**OR (95% CI)**	**Reference**
DM+CKD	11 (4-30)	[14]	Ob + CVD	3.9 (1.1-14)	[14,15]	Ob + DM	2.1 (1.3-3.4)<br>1.9 (1.2-3.1)	[14]<br>[15]
DM+COPD	3.6 (1.5-8.6)	[14]	Ob + DM	2.9 (1.8-4.5)<br>2.5 (1.6-3.9)	[14,15]	Ob + HT	2.2 (1.5-3.2)<br>2.1 (1.2-2.2)	[15]<br>[14]
DM + Ob	3.3 (2.5-4.2)<br>2.0 (1.8-2.1)	[14,15]<br>[20]	HT+ CVD	2.6 (1.2-5.6)	[14]	≥1Comorbidity	1.9 (1.7-2.0)	[20]
Ob + IS	2.8 (1.2-6.7)	[14,15]	Ob + HT	2.4 (1.6-3.6)	[14,15]	Ob	1.9 (1.5-2.5)<br>1.7 (1.4-1.9)<br>1.4 (1.2-1.6)	[15]<br>[14, 16, 20]<br>[17]
IS	2.8 (1.8-4.4)<br>1.7 (1.3-2.3)	[15]<br>[14,20]	Ob	1.9 (1.5-2.6)<br>1.7 (1.4-2.0)	[15]<br>[14,16]	DM	1.7 (1.6-1.8)<br>1.5 (1.1-2.0)<br>1.3 (1.1-1.6)	[16, 20]<br>[15]<br>[14,17]
CKD	2.4 (1.9-3.1)<br>2.0 (1.78-2.26)	[14,20]<br>[20]	DM+HT	1.8 (1.3-2.5)	[14]	IS	1.68 (1.4-1.9)	[16, 20]
DM	2.4 (2.0-2.8)<br>2.2 (1.9-2.4)	[15]<br>[14,20]	DM	1.67 (1.58-1.78)<br>1.5 (1.1-2.0)<br>1.2 (1.25-1.30)	[16]<br>[14,15]<br>[17]	Male	1.4 (1.2-1.6)	[17]
DM +HT	2.1 (1.7-2.5)	[14,20]	HT	1.4 (1.2-1.7)	[14]	HTA	1.3 (1.1-1.6)	[14]
Ob + HT	1.9 (1.6-2.4)<br>1.57 (1.5-1.6)	[14,15]<br>[20]	CKD	1.3 (1.1-1.4)	[16]	Age >65	1.3 (1.1-1.5)	[17]
Ob	1.6 (1.4-1.7)<br>1.7 (1.5-1.9)	[14,20]<br>[15]	IS	1.3 (1.1-1.5)	[16]			
COPD	1.6 (1.9-2.7)<br>1.3 (1.1-1.5)	[14]<br>[20]	Male	1.3 (1.2-1.5)	[17]			
HT	1.5 (1.3-1.7)	[14, 15, 20]	Age >65	1.3 (1.2-1.4)	[17]			
CVD	1.4 (1.1-1.7)	[14,20]						

DM= diabetes mellitus; COPD= chronic obstructive pulmonary disease; Ob = obesity; IS= inmunesuppression; CKD = Chronic kidney disease; HT = systemic hipertension; CVD= Cardiovascular disease. OR= Odds Ratio; More than one OR value may be together if all values were contained within the 95% CI (Confidence interval)

Table S4. Crude numbers of COVID-19 deaths with each comorbidity (alone or in combination with other comorbidities) per wave, in the total Mexican population (all ages)

wave	diabetes	COPD	asthma	IS	HT	Other	CVD	obesity	CKD	smoker	total COVID-19 deaths (100%)
**1 and 2**	87,537	10,228	4,063	5,170	105,883	11,896	11,657	50,894	15,803	17,875	236,944
**3**	22,309	2,450	1,115	1,325	26,067	2,882	2,809	13,119	4,322	4,239	63,738
**4**	9,628	1,458	391	871	11,967	1,752	1,776	3,367	2,969	1,965	24,861
**5**	1,607	353	73	250	2,016	426	378	393	577	357	4,496
**6**	1,140	292	56	174	1,450	264	351	333	398	259	3,127

Table S5. Mean age ± standard error at COVID-19 death in adults with each of the comorbidity combinations.

	**Wave 1 and 2**	**Wave 3**	**Wave 4**	**Wave 5**	**Wave 6**	**Wave 5 and 6**	Difference of the mean death age in years; after-before vaccines [a]	p
COPD only	72.0±0.3	72.9±0.7	77.3±1.0	80.4±1.4	80.5±1.6	80.4±1.1	8.4	0.00107
CVD only	69.3±0.5	69.0±1.0	74.3±1.4	79.5±2.5	78.5±2.5	79.0±1.8	9.7	<0.0001
HT only	68.8±0.1	68.0±0.2	74.6±0.3	77.9±0.7	78.2±0.8	78.1±0.5	9.2	<0.0001
DM-HT	67.0±0.1	66.1±0.2	70.9±0.2	74.4±0.5	74.3±0.7	74.4±0.4	7.4	<0.0001
smoker only	62.7±0.2	60.3±0.5	70.4±0.8	68.5±2.4	67.1±2.4	67.9±1.7	5.1	0.0004
**NONE**	**61.9±0.1**	**58.1±0.1**	**68.2±0.2**	**69.2±0.5**	**70.7±0.6**	**69.7±0.4**	**7.8**	<0.0001
DM only	62.3±0.1	60.4±0.2	67.2±0.4	69.9±0.9	70.6±1.2	70.2±0.7	7.8	<0.0001
DM-HT-Ob	63.2±0.1	61.2±0.3	66.0±0.5	71.9±1.7	70.9±2.0	71.5±1.3	8.2	<0.0001
HT-Ob	62.4±0.1	59.8±0.3	65.5±0.6	72.8±2.1	71.9±2.1	72.3±1.5	10.0	<0.0001
DM-HT-CKD	62.4±0.2	61.8±0.3	63.9±0.4	68.2±0.9	67.0±1.2	67.7±0.7	5.4	<0.0001
OTHER	62.8±0.3	59.2±0.7	63.6±0.9	62.3±2.0	69.0±2.4	64.8±1.5	2.0	0.0964
asthma only	57.2±0.5	54.1±1.2	61.9±2.5	67.1±5.6	61.9±6.7	64.8±4.2	7.6	0.0182
DM-Ob	58.9±0.2	55.7±0.4	62.2±0.9	65.1±3.1	65.0±5.6	65.1±2.7	6.2	0.0052
HT-CKD	58.0±0.4	56.2±0.8	60.4±1.0	65.6±2.0	58.7±3.3	63.3±1.8	5.3	0.0031
Obesity only	54.6±0.1	48.9±0.2	59.1±0.7	61.1±2.4	61.1±4.1	61.1±2.1	6.5	<0.0001
CKD only	55.3±0.5	52.8±1.0	58.0±1.4	58.8±3.3	66.5±3.7	62.2±2.5	6.9	0.0028
IS only	57.6±0.6	56.3±1.2	58.5±1.6	53.0±2.4	56.5±2.6	54.3±1.8	-3.3	0.0825

COPD= chronic obstructive pulmonary disease; CVD= Cardiovascular disease; HT = systemic hypertension; DM= diabetes mellitus; NONE= no comorbidity reported; Ob = obesity; IS= immunosuppression; CKD = Chronic kidney diseae. [a] after-before vaccine introduction was calculated by finding the diference in mean age at death during wave 1 and 2 vs wave 5 and 6

Figure S1. The specific comorbidity combinations found in the adults that died from COVID-19 during the sanitary emergency, as reported in the complete national dataset. The bracket depicts the combinations that are portrayed in figure 5 and comprise 81.5% of adult deaths. Only 0.3% of COVID-19 deaths contain no info about comorbidities in the national dataset.

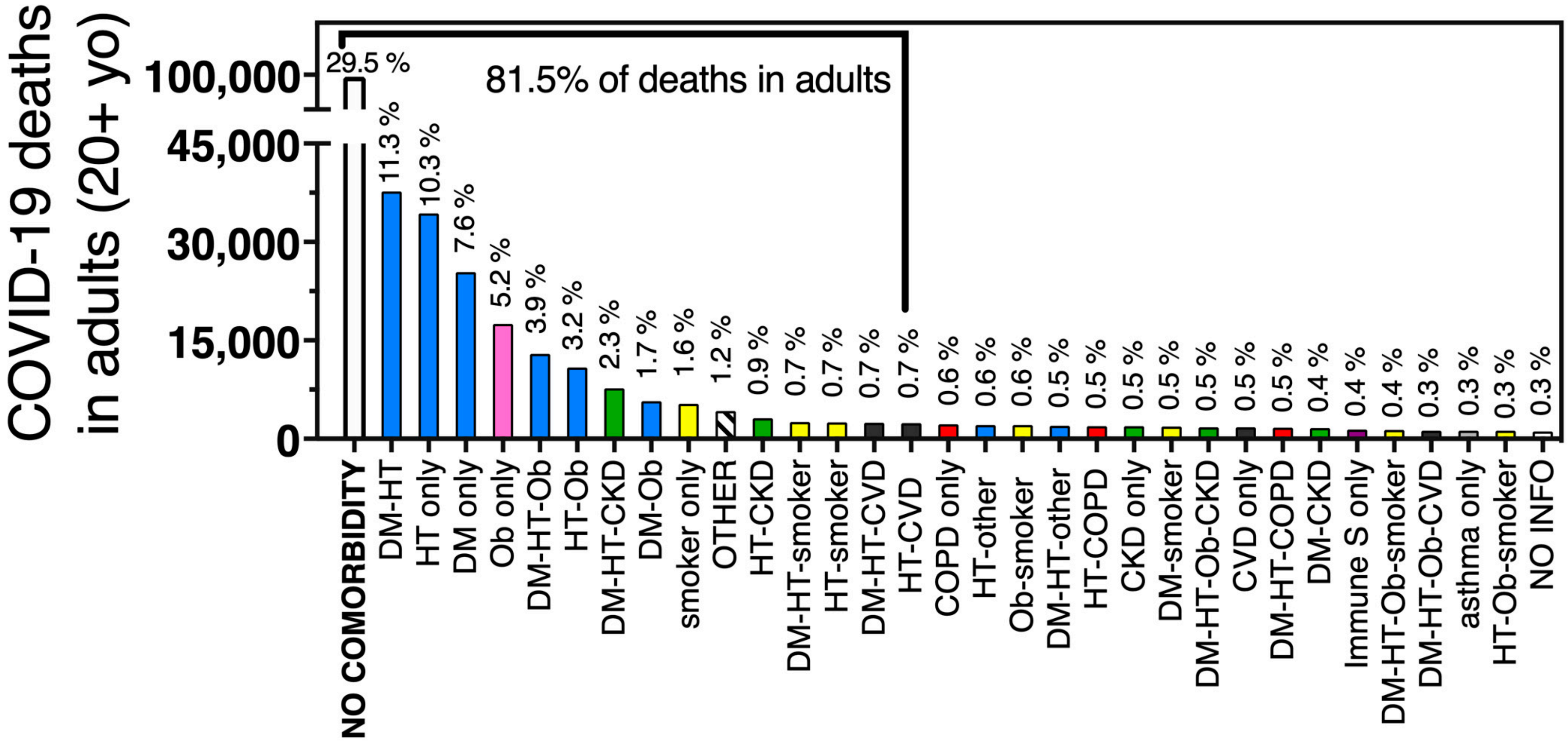

Supplement: Supplementary file 1 [file vaccines-11-01676-s001.zip › vaccines-2674884-supplementary.pdf]
